# Occupational Exposure and Health Impact Assessment of Diisocyanates in Finland

**DOI:** 10.3390/toxics11030229

**Published:** 2023-02-27

**Authors:** Pasi Huuskonen, Simo P. Porras, Bernice Scholten, Lützen Portengen, Sanni Uuksulainen, Katriina Ylinen, Tiina Santonen

**Affiliations:** 1 Finnish Institute of Occupational Health, FI-00032 Helsinki, Finland; 2 The Netherlands Organisation for Applied Scientific Research (TNO), 3508 TA Utrecht, The Netherlands; 3 Institute for Risk Assessment Sciences, Faculty of Veterinary Medicine, Utrecht University, 3584 CK Utrecht, The Netherlands

**Keywords:** biomonitoring, bronchial hyperresponsiveness, exposure assessment, exposure reconstruction, diisocyanate, health impact assessment, sensitisation, workers

## Abstract

Diisocyanates are a group of chemicals widely used in different industrial applications. The critical health effects related to diisocyanate exposure are isocyanate sensitisation, occupational asthma and bronchial hyperresponsiveness (BHR). Industrial air measurements and human biomonitoring (HBM) samples were gathered in specific occupational sectors to examine MDI, TDI, HDI and IPDI and the respective metabolites from Finnish screening studies. HBM data can give a more accurate picture of diisocyanate exposure, especially if workers have been exposed dermally or used respiratory protection. The HBM data were used for conducting a health impact assessment (HIA) in specific Finnish occupational sectors. For this purpose, exposure reconstruction was performed on the basis of HBM measurements of TDI and MDI exposures using a PBPK model, and a correlation equation was made for HDI exposure. Subsequently, the exposure estimates were compared to a previously published dose–response curve for excess BHR risk. The results showed that the mean and median diisocyanate exposure levels and HBM concentrations were low for all diisocyanates. In HIA, the excess risk of BHR from MDI exposure over a working life period was highest in the construction and motor and vehicle industries and repair sectors, resulting in estimated excess risks of BHR of 2.0% and 2.6%, and 113 and 244 extra BHR cases in Finland, respectively. Occupational exposure to diisocyanates must be monitored because a clear threshold for DI sensitisation cannot be established.

## 1. Introduction

Diisocyanates (DIs) are chemicals that contain two isocyanate functional groups (R–N=C=O, NCO). DIs are widely used in different industrial applications, most notably in the manufacturing of polyurethanes and as hardeners in industrial paints, glues, varnishes and resins [1,2]. In the European Union (EU), the estimated total tonnage of DI manufactured in and/or imported to the European Economic Area is approximately 2.5 million tons per year [3].

The most commonly used DIs include 4,4′-methylene diphenyl diisocyanate (MDI, CAS 101-68-8), toluene diisocyanate (TDI, mainly used as a mixture of 2,4-TDI and 2,6-TDI isomers CAS 26471-62-5), hexamethylene diisocyanate (HDI, CAS 822-06-0) and isophorone diisocyanate (IPDI, CAS 4098-71-9). MDI, TDI and HDI account for more than 95% of the total volume of DIs used in the EU. DIs share common toxicological properties with the functional NCO groups. The harmonised EU Classification, Labelling and Packaging (CLP) system (EU regulation 1272/2008) classifies these widely used DIs as dermal and respiratory sensitisers, and as eye, skin and respiratory irritants [4]. MDI and TDI are also classified as Carcinogenic category 2 (H351, suspected of causing cancer) [5]. The European Chemicals Agency’s (ECHA) Committee for Risk Assessment (RAC) recently published a recommendation for an occupational exposure limit (OEL) value for DIs [5]. It identified occupational asthma and bronchial hyperresponsiveness (BHR) as critical adverse health effects caused by respiratory sensitisation to DIs. However, the RAC was not able to identify a threshold value for these adverse health effects; therefore, only a dose–response was derived for BHR, and no recommendation was given for an OEL.

The use of DIs is not expected to decline in the near future because there are no suitable alternatives for most DI applications. To minimise the health risks caused by DIs, the European Commission recently adopted a restriction under the European regulation concerning the Registration, Evaluation, Authorization and Restriction of Chemicals (REACH; EC, 1907/2006) for the use of DIs. According to this restriction, DIs cannot be placed on the market or used in concentrations above 0.1% in weight unless the industrial and professional users have completed adequate professional training in the safe use of DIs [6].

Occupational exposure to DIs primarily occurs as inhalation and dermal exposure but may also occur through the gastro-intestinal tract after hand-to-mouth exposure [7], although there is no specific data on the importance of this route of exposure in case of DIs. Both the dermal and inhalation exposure routes are reported to be relevant for respiratory sensitisation [8,9,10], but the contribution of dermal exposure cannot be quantified at present [2]. TDI and HDI are relatively volatile; therefore, their concentrations can be significant at room temperature. Air levels of MDI can be high in certain conditions, for example, during spray painting. Moreover, the heating of items containing polyurethanes in, for example welding, soldering, flame cutting and sawing can produce monomeric DIs.

The monitoring of inhalation exposure to DIs is technically challenging due to their reactivity [4]. Air measurements are mainly performed outside respiratory protective equipment (RPE) and do not provide information on dermal exposure. Using human biomonitoring (HBM) data for exposure reconstruction can better highlight DI exposure through all routes of exposure, especially if workers have been exposed through skin to DIs or used RPE.

In this paper, we summarise data gathered from the Finnish Institute of Occupational Health (FIOH) databases of industrial hygiene air measurements and HBM samples, covering DI exposure in several industrial sectors in Finland during 2008–2021. These data were used for a health impact assessment (HIA) together with ECHA’s RAC dose–response curve [5] and a physiologically based pharmacokinetic (PBPK) model for exposure reconstruction of TDI and MDI exposures based on HBM measurements. For HDI exposure reconstruction, a correlation equation was used. In addition, the reconstructed exposure levels from HBM data were compared to the air monitoring exposure levels.

## 2. Materials and Methods

### 2.1. Air Measurement and Biomonitoring Data

Industrial hygiene air measurements of MDI, TDI, HDI and IPDI and urinary measurements of DI-derived diamines 4,4′-methylenedianiline (MDA), toluene diamine (TDA), hexamethylene diamine (HDA) and isophorone diamine (IPDA) were gathered for specific sectors from the Finnish registers of industrial hygiene and biomonitoring measurements between 2008 and 2021. These registers are upheld by FIOH, according to the law on the activities and funding of FIOH (STM 159/1978) and contain measurements taken by FIOH as part of its expert services to Finnish workplaces. The law on the activities and funding of FIOH (STM 159/1978) defines the information gathered in the FIOH databases and gives FIOH permission to use the collected data for research purposes. The database, which is not publicly available, conforms to the European General Data Protection Regulation (GDPR, EU, 2016/679). It consists of the original analytical results and is not a biobank, as no samples are stored. Informed consent, including consent to store the measurement results in the FIOH database, was obtained from all the workers who provided samples for analysis. The contextual information stored in the biomonitoring database concerns sample timing, sex, smoking information, job titles and company information.

The air monitoring samples included in the current data analysis were collected from the personal breathing zones of the workers. The workers’ post-shift urine samples were collected at the end of the week or after the working period that involve the use of DIs, in accordance with the FIOH sampling protocol (www.ttl.fi/biomonitorointi accessed on 29 December 2022). Information on this worker population is given in the Supplementary Materials (Table S1). The air monitoring database does not contain personal information on the workers. Information on the use of RPE was not available for air measurements or HMB data.

The air samples were analysed at FIOH using either liquid chromatography mass spectrometry (LC-MS) or liquid chromatography tandem mass spectrometry (LC-MS/MS). The limit of quantitation (LOQ) for the air measurements was 0.004 µg DI/sample for MDI, 0.002 µg DI/sample for TDI, 0.002 µg DI/sample for HDI, and 0.004 µg DI/sample for IPDI. Urinary diamines were analysed either by FIOH (before 2018, analysis method gas chromatography mass spectrometry, GC-MS) or by hired subcontractors in the United Kingdom (2018–2020, method GC-MS) or Germany (2020–2021, method LC-MS/MS). The LOQs for the urinary diamines were 0.1–1.0 µg/L for MDA, 0.1–0.6 µg/L for TDAs, 0.1–0.6 µg/L for HDA and 0.1–0.9 µg/L for IPDA. In statistical modelling, air and urinary values below LOQ were replaced with values of LOQ/2. Air concentrations for different DIs were converted to µg NCO/m^3^ using molecular weight (MW) to make the DI levels comparable. Conversion was performed using the equation:

µg NCO/m^3^ = µg DI/m^3^ × (MW NCO-groups/total MW of DI)

### 2.2. Exposure Reconstruction Using Human Biomonitoring Data

MDI and TDI exposure reconstruction was performed using a PBPK model [11]. This model can be used to estimate biomarker levels after either single or repeated exposures. Key parameters such as the absorption and elimination rates of various DIs were based on results from human controlled exposure studies. Model-based predictions of the urinary concentrations of MDA and TDA compared relatively well with the empirical results for chronic occupational exposure scenarios in a (limited) set of HBM studies from the peer-reviewed literature [12,13,14,15].

The distribution of urinary biomarker levels for each industry was estimated from the quantiles of the empirical distribution of the HBM samples using linear interpolation. The average air exposures were then estimated by Monte Carlo (MC) integration (i.e., repeatedly sampling the distribution of urinary levels, running the reverse-dosimetry algorithm, and averaging the results) using a total of 10,000 iterations.

Reverse dosimetry was performed using a Bayesian Metropolis Hastings Monte Carlo (MHMC) algorithm [16] and a weakly informative prior that constrained the reconstructed air exposure as positive. The exposure scenario used for reconstruction was based on 12 weeks of exposure for five days a week and eight hours a day and assumed that urine was collected post-shift on the last working day and that no RPE was used. The default parameter settings and uncertainty ranges are described in Scholten at al. [11], but in this paper, we report the average reconstructed air exposures obtained by Monte Carlo integration over the uncertainty ranges. The uncertainty of the PBPK model (for MDI and TDI) was not included because, based on sensitivity analyses (not shown), this uncertainty did not include the major uncertainty components related to variability in exact exposure scenarios.

As there was no PBPK model for HDI, the exposure reconstruction for HDA data was performed using the equation reported by Maître et al. [17], which is based on regression modelling of the relation between the HDI in the air and the HDA in urine for a range of HDI air concentrations (0.3–97.7 µg HDI/m^3^) and urinary HDA concentrations (1.36–27.7 µg HDA/g creatinine). The HDI and HDA concentrations were calculated using the equation:

Log_10_ (HDA) = 0.4396 × Log_10_ (HDI) + 0.4612

and for the reverse calculation of HDI concentration used in this study:

Log_10_ (HDI) = (Log_10_ (HDA) − 0.4612)/0.4396

To reduce overestimation caused by possible non-occupational background exposure, we subtracted an estimated non-occupational background level of 0.2 µg/L from the measured exposure levels used for exposure reconstruction. The background level of 0.2 µg/L was based on the available literature data on the diamine levels in the general population [18,19,20,21], but it should be noted that the information on diamine levels in the occupationally unexposed population is rather limited.

### 2.3. Health Impact Assessment

**Figure 1:**
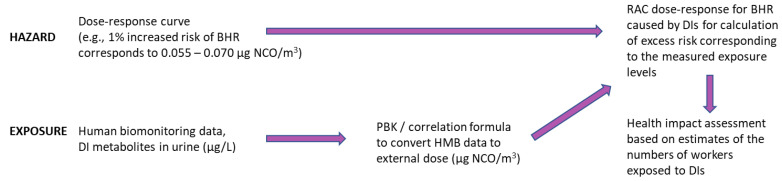
Schematic presentation of health impact assessment (HIA) using human biomonitoring (HMB) data to convert urine diisocyanate (DI) metabolite concentration to external isocyanate (NCO) exposure using a physiological based kinetic (PBK) model and a correlation formula. The dose–response curve for DI-caused bronchial hypersensitiveness (BHR) was used in HIA and provided by RAC [5].

The estimated external DI levels were compared to the exposure–excess risk relation for BHR and air NCO concentration estimated by the RAC (Figure 1). The excess risk function was estimated by interpolating the exposure-specific risk estimates provided by the RAC (Table 1) using a spline function, which considers the whole distribution of measured exposure levels. The estimated excess risks were capped at 7.5% to avoid extrapolation beyond the highest excess risk reported by the RAC (5%) (Table 1). Subsequently, an HIA was conducted using the estimated numbers of workers in Finland exposed to DIs in different sectors (Figure 1).

#### Estimated Number of Exposed Workers in Finland

The number of workers exposed to DIs in Finland (Table 2) was estimated using several data sources. As the FIOH FINJEM database (Finnish job-exposure matrix) [24] had isocyanate estimates for only a few occupations, more estimates of the percentage of the exposed workers in each occupation were calculated using the information compiled from the FIOH biomonitoring register for 2008–2016 and the number of workers obtained from Statistics Finland (2016). The sectors used in Table 2 are the result of the exposed occupational groups, as no sector-specific data were available from the FIOH registers. Construction workers included painters, plumbers and floor pavers. The estimation made as part of the EU REACH restriction proposal of workers’ exposure to Dis [25] was also utilised. As the Finnish and EU estimations of exposed workers in different occupational groups were slightly different, a mean estimate of workers exposed to Dis was calculated on the basis of the range generated from the FIOH and EU data.

The polyurethane, plastic products or furniture manufacturing sector included workers from both the plastic industry (manufacturing of polyurethane and rigid foam products) and the furniture manufacture sectors (Table 2). These sectors were combined because polyurethane and plastic are also used in furniture manufacture.

## 3. Results

### 3.1. Air Monitoring and Biomonitoring Results

Table 3 presents the results of air measurements taken in the breathing zones of the workers exposed to DIs. The results are shown separately for the four main industrial sectors: (i) construction, which mainly consisted of construction element workers, (ii) motor vehicle manufacturing and repair, which consisted mostly of vehicle painters, but also boat builders, welders and mechanics, (iii) manufacture of polyurethane products, which mainly consisted of production workers in the plastics and insulation industry and in paint factories, and (iv) assemblers of industrial products, which mainly consisted of workers from shoe, window, door and machine manufacturing.

The highest measured MDI and HDI levels were in the motor vehicle manufacturing and repair sector, whereas TDI exposure was highest in the manufacture of polyurethane, plastic products or furniture, and among assemblers of industrial products. Due to the low number of measurements, the IPDI data were not further stratified by industrial sectors. The number of air monitoring samples exceeding Finnish short-term (15 min) OEL for DIs (35 µg NCO/m^3^) were for MDI *n* = 2 (0.5%), TDI *n* = 0 (0%), HDI *n* = 2 (1.4%) and IPDI *n* = 0 (0%).

Table 4 presents the urinary DI metabolite results. The HBM samples are from the same sectors as the DI air measurement data. The highest MDA levels were among assemblers of industrial products, while HDA was the highest in the motor vehicle manufacturing and repair sector, and the highest TDA concentrations were measured in the manufacture of polyurethane, plastic products or furniture. As less than 10% of the IPDA results could be quantified reliably, these were not further stratified into specific sectors. In fact, rather a large proportion of the HBM data could not be quantified (i.e., data were below the LOQ) (Table 4). Creatinine-corrected HBM results are provided in the Supplementary Materials (Table S2).

Because of the small overall sample numbers, annual data per sector cannot be provided. However, the whole dataset was analysed to see if there was any declining trend over the follow-up period. No trend was observed).

### 3.2. Health Impact Assessment

Table 5 presents the results of exposure reconstruction from the HBM data, and the corresponding reconstructed urinary levels are presented in the Supplementary Materials, Table S3. Exposure was not reconstructed for TDI and HDI in construction and TDI in the motor vehicle sector due to the low number of total measurements and/or measurements above LOQ. The excess risk of BHR over a working life period caused by DI exposure in the various sectors was calculated (Table 6). In general, the excess risk was highest for MDI in the motor and vehicle industry and repair sector, where an excess BHR risk of 2.6% was estimated. This indicates that in this Finnish sector, the expected excess number of BHR cases is 244. For HDI, which is typically used in the motor and vehicle industry and the repair sector pose an excess risk of 1.5%, which indicates 144 excess BHR cases among Finnish workers. In the occupational groups that manufactured polyurethane, plastic products or furniture and among the assemblers of industrial products, the highest BHR risk was estimated for MDI exposure, 2.1% and 1.6% resulting in 35 and 24 extra BHR cases, respectively.

## 4. Discussion

We summarised the data gathered from the FIOH databases of industrial hygienic air measurements and HBM samples, covering DI exposure in several industrial sectors in Finland in 2008–2021. After exposure reconstruction, HBM data were utilised for the human HIA of excess risk of BHR according to ECHA’s RAC dose–response curve [5].

### 4.1. Occupational Exposure to Diisocyanates in Finland

In the Finnish data, the mean and median air exposure levels and HBM concentrations were low for all DIs in all the occupational sectors (Table 3 and Table 4). However, the exposure distribution was highly skewed, resulting in high P95 concentrations driven by a few high exposures in some sectors in both the air measurements and the HBM data. A large proportion of the analysed air monitoring and HBM results were below the LOQ, suggesting low exposure. However, it should be noted that in many cases, all four DIs or DI metabolites were measured, although only one or two of them were actually used in the workplace. Unfortunately, it was not possible to extract the information on the exact DIs in use at the workplace from the FIOH biomonitoring database. For example, in motor vehicle manufacturing and repair, HDI is typically used in paints and coatings, and MDI exposure may occur in spray application of polyurethane foam, but TDI occurs less commonly in this sector. Nevertheless, all four DIs were analysed, which obviously affected the amount of data below the LOQ. The current Finnish short-term (15 min) OEL for isocyanates is 35 µg NCO/m^3^. No 8 h time-weighted average (TWA) OELs have been set for DIs in Finland. The European Commission’s Advisory Committee on Safety and Health at Work (ACSH) has recently proposed a binding occupational exposure limit of 10 µg/m^3^ (measured as NCO, total reactive isocyanate groups), reducing it to 6 µg/m^3^ from 1 January 2029 [26]. Twenty measurements (2.9% of all measurements) exceeded the limit of 10 µg NCO/m^3^ and 34 measurements (4.9%) exceeded 6 µg NCO/m^3^. Although the Finnish mean/median air monitoring data levels were well below these levels in the analysed period, even these levels may not be without a risk, as it is currently not possible to identify a threshold for the sensitising properties of DIs [5].

However, it should be noted that in most of the known high exposure-level work tasks, such as spray painting and polyurethane blasting, the workers used RPE, and the air samples were collected outside the respirator. When RPE is used, biomonitoring can give a more realistic overview of the real exposure. Biomonitoring can also give information on exposure via other routes, including via dermal absorption or ingestion (due to hands-to-mouth exposure). Dermal exposure has shown to contribute to the sensitisation risk of DIs [9,10]. There are no biological limit values available in Finland for DIs. In the USA, the American Conference of Governmental Industrial Hygienists (ACGIH) has set biological exposure indices (BEI) for TDI (5 µg/g creatinine, measured as the sum of urine 2,4- and 2,6-TDA) and for HDI (15 µg/g creatinine, measured as urine HDA) [27,28]. These corresponded to exposure to threshold limit values (TLVs) of 0.001 ppm and 0.005 ppm (0.007 and 0.034 mg/m^3^ for TDI and HDI, respectively) when sampled at the end of the shift. Similarly, the Deutsche Forschungsgemeinschaft (DFG) has given a biological tolerance (BAT) value of 15 µg/g creatinine, measured as urinary HDA, for HDI and a BAT value of 5 µg/g creatinine, measured as the sum of urinary 2,4- and 2,6-TDA, for TDI [29,30].

In addition to the RAC restriction proposal on DIs [5], occupational exposure to DIs has also been recently reviewed by Scholten et al. [1] and Rother and Schlüter [2]. The DI levels reported in the literature indicate that mean/median DI exposure in Europe is below 30 µg NCO/m^3^. However, in spraying applications, the highest HDI air concentrations have been measured during spray painting and coating (>400 µg NCO/m^3^) [7,31,32,33,34,35]. In addition, in studies with spray foam applications, MDI levels have indicated altering ranges of exposure from <LOQ peaking up to 700 µg NCO/m^3^ [7,15,36,37,38]. The most studied application of TDI is the manufacture of foams, in which air levels have had varying ranges, from <0.1 to 140 µg NCO/m^3^ [7,12,39,40,41,42,43].

In the construction sector, the Finnish HBM data (Table 4) for MDI suggest similar exposure to earlier published biomonitoring data, GM being 0.3 µg MDA/L [14,44]. In the manufacture of polyurethane products, the FIOH database data suggest lower MDI exposure than the data published on this sector: of five published studies [19,45,46,47] in this sector, two report higher exposure levels. In the Finnish dataset (Table 4), the GM and median levels for MDA were 0.3 and 0.4 µg MDA/L, respectively, whereas Robert et al. [21] reported a mean level of 1.6 µg/L in France and Sennbro et al. [45] reported a median of 2 µg/L in Sweden. Of the eight studies published on TDI among assemblers of industrial products, at least four [12,42,46,48] report much higher average amine levels (0.3 to 18 µg TDA/L) than observed in the Finnish dataset (GM 0.1 µg/L, Table 4).

The HDA levels reported in the motor vehicle manufacturing and repair sector in the UK have been lower (GM 0.1 [49] and P90s 1.8–4.0 µg/L [50]) than those in the Finnish data (GM 0.4 µg/L HDA range <LOQ–44.4 µg/L, Table 4). However, data from the Netherlands [51] showed a higher median level of 21.5 µg HDA/g creatinine (approx. 29 µg HDA/L when a mean urine creatinine concentration of 1.36 g/L [52] was used) and up to 150.2 µg HDA/g creatinine (approx. 204 µg HDA/L).

### 4.2. Exposure Reconstruction, Health Impact Assessment and Related Uncertainties

This study provided both measured air levels and reconstructed air levels based on HBM data. The levels (Table 3 and Table 5) did not generally overlap. This could not be explained by the potential use of personal protective equipment, because in all but one case (i.e., TDI among assemblers of industrial products), exposure levels were higher when based on HBM data than when based on direct air measurements. One possible explanation is dermal exposure, which may become relevant especially in exposure scenarios using MDI, which is less volatile than the other two DIs. The difference between estimated air levels and HBM data could perhaps also be (partly) explained by differences in sampling times; for example, Scholten et al. [11] demonstrated the importance of exposure scenarios on biomonitoring levels measured after the exposure. The specific information on the timing and frequency of exposure brings some uncertainty to the assessment. In the case of infrequent tasks, this may result in either over- or underestimation of health risks depending on whether the sample has been taken (as instructed) after the tasks involving exposure to DIs or (incorrectly) after the working shift, which involves no clear DI-related tasks. FIOH’s sampling guidance advises occupational health services to perform biomonitoring of workers who use DIs in their work. These data may, however, underestimate rather than overestimate Finnish DI exposure, because these workplaces may represent companies with good occupational health services and better awareness of the health risks of DIs. In addition, the process of exposure reconstruction also has inherent limitations. However, although the applied PBPK model is not yet fully calibrated, it compared relatively well with the published aggregated data used earlier to, for example, set BEI and BAT values for TDI [29,30]. For HDI, a correlation formula by Maitre et al. [17] was used. The approximate range of HDI concentrations in the study of Maitre et al. was 0.2–49.0 µg NCO/m^3^, and the urinary biomonitoring HDA concentrations ranged from 1.4 to 28.0 µg/g creatinine. Values outside these concentration ranges of the equation depended heavily on extrapolation. In addition, correlation has been established for air HDI monomer and urinary HDA. This may result in underestimation of the exposure to reactive NCO groups in HDI prepolymers, which are not reflected as elevated HDA levels.

The Finnish air monitoring data (Table 3) showed up to a 3% excess risk of BHR over the working life period (Table 1) for GM and median TDI exposure in Finnish manufacturing of polyurethane, plastic products or furniture. However, at the P95 levels of DI, the air monitoring data in nearly all sectors exceeded the highest risk of BHR (5%) presented in the RAC dose–response [5].

In the last 10 years, four cases of occupational asthma due to DI exposure have been diagnosed on average annually in Finland [53]. This means on average 160 asthma cases in 40 years, which is a low number compared to the calculated BHR cases in Table 6. These asthma cases have been observed among stationary plant and machine operators, construction workers, assemblers of industrial products, metal and machinery workers, and personal service workers [54]. The estimated annual number of DI-related occupational asthma incidences in the EU is over 5000 [2].

Although there may be some underdiagnosis of DI-caused BHR/asthma in Finland, it needs to be recognised that the risk assessment and HIA performed in this study had several related uncertainties. The uncertainties related to the RAC dose–response were pointed out in the RAC opinion [5]. The exposure risk relations obtained from two independent studies [22,23] on work life-long exposure were adjusted by multiplying the calculated risks by a factor of 2. In addition, calculation over a working life period is relatively conservative as it has been indicated that one to two years after the onset of exposure, the risk levels off. The dose–response did not consider the effects of peak exposure or dermal exposure, although these are also relevant for respiratory sensitisation.

There are also significant uncertainties related to the highly skewed nature of exposure data on the risks and on human HIA. As can be seen in Table 4, most of the exposure data were below the LOQ. For exposure reconstruction and risk assessment, LOQ divided by two was used in these cases. This is likely to result in overestimating the risk, especially considering that in many cases, all three DI metabolites were measured although the exposure may have been to only one or two specific DIs. More sensitive analytical techniques could have diminished the uncertainty related to this approach. To reduce the impact of background levels and levels <LOQ to risk assessment, we used a level of 0.2 µg/L as an estimate of background levels and subtracted it from the measured exposure levels used for exposure reconstruction. We noted that the information on diamine levels in occupationally unexposed population is currently rather limited. Sennbro et al. [18] reported urinary MDA levels of <0.05–3 µg/L with a P95 level of 0.3 µg/L among 120 occupationally non-exposed adults. The range and P95 levels were <0.1–0.4 µg/L and 0.1 µg/L for 2,4-TDA, respectively, and <0.1–0.2 µg/L and 0.1 µg/L for 2,6–TDA [18]. In Finland, Rosenberg et al. [19] reported the range of 0.01–0.14 µg/L for urinary MDA and ND-0.16 µg/L to urinary TDA (both isomers) in a small occupationally unexposed group of workers (*n* = 10). The levels in the range of <0.1–0.87 µg/L (range) for urinary MDA have been reported also by Robert et al. [21]. The National Health and Nutrition Examination Survey (NHANES) [20] reported median and P95 levels of 0.05 and 0.367 µg/L for urinary MDA in U.S. adults, whereas in case of 2,4-TDA, median levels were below the LOD (0.64 µg/L) and P95 was 0.895 µg/L (all 2,6-TDA levels were below LOD). No such information was available on urinary HDA levels. Based on these data, we considered 0.2 µg/L as the best estimate on the background levels but emphasise the need for more recent, good quality data on the general population urinary diamine levels.

The arithmetic mean values in each sector were affected by a few high values, which were above the levels for which ECHA’s RAC [5] has a derived dose–response. The highly skewed nature of the dataset is one of the reasons why the use of single exposure determinants for exposure reconstruction and health risk assessments was not considered appropriate and whole exposure distribution was used instead. We estimated the population distribution of urinary biomarker levels by linear interpolation of the reported quantiles because the strongly skewed distributions of the measured urinary biomarker values appeared to be incompatible with the single parametric (e.g., log-normal) distributions. To avoid extrapolation outside the ECHA RAC dose–response [5], the risk was capped to an excess risk level of 7.5%, limiting the contribution of the few highly exposed workers to the total risk.

One important uncertainty in the human HIA was related to the number of exposed workers. In this HIA, we used estimated figures of exposed workers for each sector. However, these estimates are generally difficult to extract. In this case, we used the average numbers of exposed workers estimated on the basis of the FIOH FINJEM database, the FIOH biomonitoring register, and the number of workers in Statistics Finland records, as well as estimates made as part of REACH diisocyanate restriction [25]. Because of the uncertainties related to these estimations, they must be interpreted with caution. Although the numbers of BHR cases were affected by the estimation of a high number of exposed workers it, however, did not affect the excess risk of BHR percentages presented in Table 6.

In Table 6, we calculated the human health impacts for each individual DI measured in the given sector. It must be noted that this assumes that the measured urinary metabolite levels were representative of all workers in the given industry sector, not only those who were exposed to a particular DI species. However, this is not likely to be the case: although in some tasks, co-exposure to two different DIs is possible, and in many cases, different DIs are used in totally different tasks. For example, in the motor vehicle manufacturing and repair sector HDI is typically used to spray coat vehicles, whereas MDI exposure may occur when polyurethane foam is used as insulation material, for example. These tasks may be performed by different workers. As we did not estimate the number of workers performing separate tasks and only the total number of potentially exposed workers in each sector, adding up excess BHR cases for each individual DI to calculate the total burden of disease may result in some double counting and overestimating the risk. If we assume that 2/3 of the workers perform HDI painting (which is a more common task in the motor vehicle sector) and 1/3 perform MDI-related tasks, this means that 93 BHR cases were caused by HDI and 81 cases were caused by MDI: a total of 174 combined excess BHR cases over a 40-year work life period. The same applies to the polyurethane, plastic products or furniture manufacturing sector, which includes tasks related to manufacturing polyurethane products, foams and furniture. Whereas in the polyurethane industry, the main DI in use is TDI, in the furniture sector, some workers are mainly exposed to MDI when using MDI-based glues. Considering that half of the total 1700 workers can be assumed to work in the polyurethane industry and half can be assumed to work in the furniture industry, the real numbers of excess BHR cases in this sector might be up to two times lower. However, as discussed earlier, there are significant uncertainties related to the number of exposed workers per sector.

The advantages of performing risk assessments or HIA by using HBM data instead of air monitoring data are related to RPE use and skin exposure. The only way to ensure RPE effectiveness when working with DIs is to take HBM samples, as air concentration measurements would overestimate personal exposure. In addition, skin exposure to DIs contributes to the risk of BHR and can be monitored by HBM. For example, in the motor and vehicle manufacture and repair sector, many paints and coatings contain HDI, which cannot be applied without protective clothing and RPE. However, it remains challenging to relate reconstructed exposure levels based on HBM to a risk relationship based on inhalation exposure.

Although the observed exposure levels were generally low in comparison to the available OELs for DIs, high exposures may occur in some tasks, resulting in an elevated risk of BHR. The highest numbers of excess BHR cases were estimated to occur in the construction sector and motor vehicle manufacturing and repair, which also showed the highest numbers of exposed workers. In the future, the sensitivity of the analysis methods in DI screening should enable the detection of even lower HBM levels of these compounds to efficiently monitor exposure. This requires also better characterisation of general population background levels from environmental sources and consumer products, as the current information on background levels in adult population is rather limited. The exposure levels of DIs may also be lower in the future, as the new EU regulation/restriction (EC 2020/1149) [6] on working with DIs enters into force. However, occupational exposure to DIs still needs to be monitored because a threshold limit value for DI-caused sensitisation cannot be established.

## Figures and Tables

**Table 1:** Excess risk over working life period of bronchial hyperresponsiveness or diisocyanate asthma [5].

Excess Risk Over Working Life Period	Exposure–Response Relations in μg/m^3^ NCO in Air ^1^
0.1%	<0.025
0.5%	0.027–0.040
1%	0.055–0.070
2%	0.12–0.19
3%	0.22–0.33
4%	0.40–0.48
5%	>0.67

^1^ Based on the studies by Collins et al. [22] and Pronk et al. [23].

**Table 2:** Estimated number of workers exposed to diisocyanates in certain occupation groups in Finland.

Exposed Occupation Groups	Estimated Total Number of Workers (*n*)	Estimated Number of Exposed Workers (Range, %)	Mean Estimated Number of Exposed Workers (*n*, %)
Construction workers, finishing tasks	25,300	2500–9000 ^1^ (10–36)	5700 (23)
Motor vehicle service and repair workers (painting)	22,000	8100 ^1^–10,600 ^2^ (37–48)	9300 (42)
Assemblers of machinery and electric devices	14,600	1500 ^1^ (10)	1500 (10)
Furniture manufacture workers	5300	500 ^1^–1000 ^2^ (10–19)	800 (15)
Process workers in plastic product industry	4000	800 ^2^–1000 ^1^ (20–25)	900 (23)
Total	71,200		18,200 (26)

^1^ FINJEM/FIOH estimation; ^2^ Estimation used in REACH diisocyanate restriction [25].

**Table 3:** Diisocyanate air monitoring data measured in breathing zones of workers in specific sectors from Finnish occupational studies in 2008–2021.

Sector	Diisocyanate	Air Monitoring Results (µg/m^3^ NCO) ^1^			
	(N of Air Samples)	GM	Median (P50)	P95	Max
Construction	MDI (*n* = 37) ≥LOQ *n* = 7 (19%)	0.01	<LOQ	0.2	0.9
	TDI (*n* = 3) ≥LOQ *n* = 1 (33%)	-	-	-	0.06
	HDI (*n* = 6) ≥LOQ *n* = 0 (0%)	<LOQ	-	-	<LOQ
Motor vehicle manufacturing and repair	MDI (*n* = 60) ≥LOQ *n* = 27 (45%)	0.05	<LOQ	23.8	90.5
	TDI (*n* = 7) ≥LOQ *n* = 4 (57%)	0.2	0.2	-	8.6
	HDI (*n* = 81) ≥LOQ *n* = 63 (78%)	0.2	0.2	8.1	153.0
Manufacture of polyurethane, plastic products or furniture	MDI (*n* = 124) ≥LOQ *n* = 75 (61%)	0.04	0.02	0.8	12.4
	TDI (*n* = 56) ≥LOQ *n* = 40 (71%)	0.3	0.3	8.0	22.3
	HDI (*n* = 13) ≥LOQ *n* = 4 (31%)	0.02	<LOQ	-	0.03
Assembly of industrial products	MDI (*n* = 154) ≥LOQ *n* = 76 (49%)	0.04	<LOQ	1.1	30.7
	TDI (*n* = 51) ≥LOQ *n* = 25 (49%)	0.1	<LOQ	11.6	25.5
	HDI (*n* = 36) ≥LOQ *n* = 11 (31%)	0.02	<LOQ	0.5	1.0
All air monitoring samples ^2^	MDI (*n* = 390) ≥LOQ *n* = 191 (49%)	0.03	<LOQ	1.2	90.5
	TDI (*n* = 118) ≥LOQ *n* = 71 (60%)	0.2	0.1	8.2	25.5
	HDI (*n* = 138) ≥LOQ *n* = 80 (58%)	0.1	0.03	6.6	153.0
	IPDI (*n* = 52) ≥LOQ *n* = 35 (67%)	0.03	0.03	0.3	1.5

GM = geometric mean, LOQ = limit of quantitation, NCO = isocyanate functional group, P = percentile. ^1^ Values below LOQ were replaced with values of LOQ/2; ^2^ Data above Finnish short-term (15 min) OEL for DIs, 35 µg NCO/m^3^: MDI *n* = 2 (0.5%), TDI *n* = 0 (0%), HDI *n* = 2 (1.4%), IPDI *n* = 0 (0%).

**Table 4:** Human biomonitoring data on diisocyanates for specific sectors from Finnish occupational studies in 2008–2021.

Sector	Metabolite	Urinary Biomonitoring Results (µg/L) ^1^			
	(*N* of Urine Samples)	GM	Median (P50)	P95	Max
Construction	MDA (*n* = 48) ≥LOQ n = 35 (73%)	0.3	0.4	1.4	4.0
	TDA (*n* = 8) ≥LOQ *n* = 0 (0%)	<LOQ	<LOQ	-	<LOQ
	HDA (*n* = 8) ≥LOQ *n* = 3 (38%)	0.9	<LOQ	-	135.2
Motor vehicle manufacturing and repair	MDA (*n* = 54) ≥LOQ *n* = 10 (19%)	0.3	<LOQ	1.1	19.6
	TDA (*n* = 40) ≥LOQ *n* = 4 (10%)	0.2	<LOQ	-	0.3
	HDA (*n* = 43) ≥LOQ *n* = 8 (19%)	0.4	<LOQ	15.7	44.4
Manufacture of polyurethane, plastic products or furniture	MDA (*n* = 82) ≥LOQ *n* = 24 (29%)	0.3	<LOQ	1.3	6.8
	TDA (*n* = 70) ≥LOQ *n* = 11 (16%)	0.2	<LOQ	0.3	10.4
	HDA (*n* = 35) ≥LOQ *n* = 8 (23%)	0.4	<LOQ	16.6	20.0
Assembly of industrial products	MDA (*n* = 177) ≥LOQ *n* = 47 (27%)	0.2	<LOQ	0.5	81.5
	TDA (*n* = 98) ≥LOQ *n* = 8 (8%)	0.1	<LOQ	0.3	3.3
	HDA (*n* = 92) ≥LOQ *n* = 14 (15%)	0.2	<LOQ	2.8	34.7
All biomonitoring samples	MDA (*n* = 366) ≥LOQ *n* = 119 (33%)	0.2	<LOQ	1.2	81.5
	TDA (*n* = 222) ≥LOQ *n* = 25 (11%)	0.2	<LOQ	0.3	10.4
	HDA (*n* = 181) ≥LOQ *n* = 33 (18%)	0.3	<LOQ	9.6	135.2
	IPDA (*n* = 155) ≥LOQ *n* = 13 (8%)	0.1	<LOQ	0.6	16.3

GM = geometric mean, HDA = hexamethylene diamine, IPDA = isophorone diamine, LOQ = limit of quantitation, MDA = 4,4′-methylenedianiline, P = percentile, TDA = toluene diamine. ^1^ Values below LOQ were replaced with values of LOQ/2.

**Table 5:** Exposure reconstruction from biomonitoring data based on diisocyanate exposure in specific sectors in Finland. The exposure reconstruction was performed using a PBPK model for MDI and TDI and a regression modelling equation for HDI. All measured median levels were <LOQ; therefore, no reconstructed P50 levels are presented. The corresponding reconstructed urinary levels are presented in the Supplementary Materials, Table S3.

Sectors	Diisocyanate	Exposure Reconstruction (µg NCO/m^3^)		
		GM	AM	P95
Construction ^1^	MDI	0.0003	0.28	0.93
Motor vehicle manufacturing and repair ^1^	MDI	0.06	0.68	0.73
	HDI	0.0001	9.7	32.3
Manufacture of polyurethane, plastic products or furniture	MDI	0.0004	0.35	1.21
	TDI	0.0001	0.21	0.23
	HDI	0.0001	4.61	37.9
Assemblers of industrial products	MDI	0.0002	1.77	0.25
	TDI	2 × 10^−7^	0.08	0.08
	HDI	3 × 10^−16^	3.75	2.49

AM = arithmetic mean, GM = geometric mean, P = percentile. ^1^ Exposure was not reconstructed for TDI and HDI in construction and TDI in the motor vehicle sector due to the low number of total measurements and/or measurements above LOQ.

**Table 6:** Exposure reconstruction from biomonitoring data and estimated excess risk over a working life period of developing bronchial hyperresponsiveness (BHR) based on diisocyanate exposure in specific sectors in Finland. The excess risk estimations were based on RAC dose–responses for diisocyanates that cause BHR.

Sectors (Estimated Number of Exposed Workers)	Diisocyanate	Excess Risk (%)	Number of Excess Cases of BHR
Construction (*n* = 5700) ^1^	MDI	2.0	113
Motor vehicle manufacturing and repair (*n* = 9300) ^1^	MDI	2.6	244
	HDI	1.5	144
Manufacture of polyurethane, plastic products or furniture (*n* = 1700)	MDI	2.1	35
	TDI	0.7	12
	HDI	1.5	26
Assembly of industrial products (*n* = 1500)	MDI	1.6	24
	TDI	0.6	9
	HDI	0.7	11

^1^ Due to the low number (0–4) of total measurements and/or measurements above LOQ, TDI and HDI in construction and TDI in the motor vehicle sector were not included in health impact assessment.

## Data Availability

The data presented in this article are not publicly available.

## Supplementary Materials

The following supporting information can be downloaded at: https://www.mdpi.com/article/10.3390/toxics11030229/s1, Table S1. Description of occupational study population of human biomonitoring samples of diisocyanate exposure; Table S2. Human biomonitoring data on diisocyanates for specific sectors from Finnish occupational studies in 2008–2021. Concentrations are given in unit of μg/g creatinine; Table S3. Exposure reconstruction of biomonitoring data based on diisocyanate exposure in specific sectors in Finland. The exposure reconstruction was performed using a PBPK model for MDA and TDA, and a regression modelling equation for HDA. All measured median levels were <LOQ, therefore, no reconstructed P50 levels are presented.
