# Supplementary material for: Occupational Exposure and Health Impact Assessment of Diisocyanates in Finland
Source: Toxics. 2023 Feb 27;11(3):229. doi: 10.3390/toxics11030229 (PMC10052111; doi:10.3390/toxics11030229)
Supplement: Supplementary file 1 [file toxics-11-00229-s001.zip › toxics-2163526-supplementary.pdf]

Supplementary Materials: Occupational Exposure and Health Impact Assessment of Diisocyanates in Finland

Pasi Huuskonen, Simo P. Porras, Bernice Scholten, Lützen Portengen, Sanni Uuksulainen, Katriina Ylinen and Tiina Santonen

Table S1. Description of occupational study population of the human biomonitoring samples on diisocyanate exposure.

Diisocyanate Metabolite (*n*)	Range of Age (a)	Gender	Smoking
MDA<br>(*n* = 366)	19–64	98 women<br>268 men	211 non-smokers<br>130 smokers<br>24 not available
TDA<br>(*n* = 222)	21–64	62 women<br>160 men	138 non-smokers<br>74 smokers<br>9 not available
HDA<br>(*n* = 181)	21–64	52 women<br>129 men	112 non-smokers<br>60 smokers<br>9 not available
IPDA<br>(*n* = 155)	21–64	47 women<br>108 men	100 non-smokers<br>50 smokers<br>5 not available

HDA = hexamethylene diamine, IPDA = isophorone diamine, MDA = 4,4′-methylenedianiline, TDA = toluene diamine.

Table S2. Human biomonitoring data on diisocyanates for specific sectors from Finnish occupational studies during 2008–2021. Concentrations are given in unit of μg/g creatinine.

Sector	Metabolite	Urinary Biomonitoring Results (μg/g Creatinine)[1]			
	(N of Urine Samples)	GM	Median (P50)	P95	Max
Construction	MDA (48)<br>≥LOQ *n* = 35 (73%)	0.4	0.4	1.9	3.0
	TDA (8)<br>≥LOQ *n* = 0 (0%)	<LOQ	<LOQ	-	<LOQ
	HDA (8)<br>≥LOQ *n* = 3 (38%)	1.1	<LOQ	-	124.5
Motor vehicle manufacturing and repair	MDA (54)<br>≥LOQ *n* = 10 (19%)	0.3	<LOQ	1.3	17.4
	TDA (40)<br>≥LOQ *n* = 4 (10%)	0.2	<LOQ	-	1.2
	HDA (43)<br>≥LOQ *n* = 8 (19%)	0.4	<LOQ	13.3	50.3
Manufacturing polyurethane products, polyurethane industry and rigid foam production	MDA (82)<br>≥LOQ *n* = 24 (29%)	0.2	<LOQ	1.5	3.9
	TDA (70)	0.2	<LOQ	0.9	8.5

	≥LOQ n = 11 (16%)				
	HDA (35) ≥LOQ n = 8 (23%)	0.5	<LOQ	10.3	13.7
Assemblers of industrial products	MDA (177) ≥LOQ n = 47 (27%)	0.2	<LOQ	1.2	21.8
	TDA (98) ≥LOQ n = 8 (8%)	0.1	<LOQ	0.7	2.7
	HDA (92) ≥LOQ n = 14 (15%)	0.2	<LOQ	4.1	77.6
All biomonitoring samples	MDA (366) ≥LOQ n = 119 (33%)	0.3	<LOQ	1.5	21.8
	TDA (222) ≥LOQ n = 25 (11%)	0.2	<LOQ	0.9	8.5
	HDA (181) ≥LOQ n = 33 (18%)	0.3	<LOQ	9.9	124.5
	IPDA (155) ≥LOQ n = 13 (8%)	0.1	<LOQ	0.4	9.6

GM = geometric mean, HDA = hexamethylene diamine, IPDA = isophorone diamine, LOQ = limit of quantitation, MDA = 4,4′-methylenedianiline, P = percentile, TDA = toluene diamine. [1]Values below LOQ were replaced with values of LOQ/2.

Table S3. Exposure reconstruction of biomonitoring data based on diisocyanate exposure in specific sectors in Finland. The exposure reconstruction was performed using a PBPK model for MDA and TDA, and a regression modelling equation for HDA. All measured median levels were <LOQ, therefore, no reconstructed P50 levels are presented.

Sectors	Diamine	Urinary Biomonitoring (μg DI/L)		
		**GM**	**AM**	**P95**
**Construction***	MDA	0.0004	0.34	1.16
Motor vehicle manufacturing and repair*	MDA	0.08	0.78	0.90
	HDA	0.06	3.05	15.4
Manufacture of polyurethane, plastic products or furniture	MDA	0.0005	0.44	1.50
	TDA	0.0001	0.32	0.34
	HDA	0.06	2.57	16.5
Assemblers of industrial products	MDA	0.0003	2.2	0.31
	TDA	3×10^{-7}	0.11	0.11
	HDA	5×10^{-7}	1.28	4.98

AM = arithmetic mean, GM = geometric mean, HDA = hexamethylene diamine, MDA = 4,4′-methylenedianiline. P = percentile, TDA = toluene diamine. * Exposure was not reconstructed for TDA and HDA in construction and TDA in the motor vehicle sector due to the low number of total measurements and/or measurements above LOQ.
